# Variation in traditional knowledge of culturally important macromycete species among three indigenous communities of Oaxaca, Mexico

**DOI:** 10.1186/s13002-024-00679-8

**Published:** 2024-03-22

**Authors:** Alexanders López-García, Marko Gómez-Hernández, Etelvina Gándara

**Affiliations:** 1 Centro Interdisciplinario de Investigación para el Desarrollo Integral Regional, Unidad Oaxaca, Instituto Politécnico Nacional, Hornos No. 1003, CP 71230 Santa Cruz Xoxocotlán, Oaxaca Mexico; 2 CONAHCYT. Centro Interdisciplinario de Investigación para el Desarrollo Integral Regional, Unidad Oaxaca, Instituto Politécnico Nacional, Hornos No. 1003, CP 71230 Santa Cruz Xoxocotlán, Oaxaca Mexico; 3 Facultad de Ciencias Biológicas, Benemérita Universidad Autónoma de Puebla, Av. San Claudio S/N Col. Ciudad Universitaria, CP 72592 Puebla, Mexico

**Keywords:** Ethnomycology, Wild mushrooms, Traditional knowledge, Edible mushrooms, Medicinal mushrooms, Indigenous nomenclature, Chatinos, Chontales, Chinantecos

## Abstract

**Background:**

For centuries, wild mushrooms have been a forest resource of significant cultural value in several ethnic groups worldwide. In Mexico, extensive traditional knowledge on the use of fungal resources has been developed and deeply rooted. Mexico is the second country in the world in which the most species of wild mushroom are consumed, and it is considered a pioneer in ethnomycology. Nonetheless, there are still many indigenous groups in this country that have not been studied from an ethnomycological approach. The present study aimed to record the traditional knowledge on wild mushrooms in three indigenous groups of the state of Oaxaca, Mexico, and assess the variation in this knowledge within and across the studied groups.

**Methods:**

The data were recorded from April to October 2022 within three communities belonging to the indigenous groups Chatino, Chontal, and Chinanteco. Through 84 interviews, information related to their knowledge of wild mushrooms was obtained. The cultural significance index of wild edible mushrooms was calculated for each community. Regression analyses, analysis of variance and covariance, *t* test, and non-metric multidimensional scaling analysis were performed to assess the distribution of traditional knowledge in the communities.

**Results:**

A total of 32 culturally important mushroom species were recorded for the three indigenous groups (30 edible, 2 medicinal); 23 used by Chatinos, 16 by Chontales, and 6 by Chinantecos. Only Chatinos and Chinantecos use wild mushrooms in medicine. The cultural significance of wild edible mushrooms differed among groups. Traditional knowledge about wild mushrooms declines when the level of schooling increases and age decreases, especially in the Chatino group. This knowledge distributes more homogeneously in the Chontal and Chinanteco groups. Their age determines the difference in knowledge between men and women.

**Conclusion:**

Documenting how traditional knowledge differs among ethnic groups is relevant for preserving cultural and biological diversity. Factors such as level of schooling and age can affect traditional knowledge of wild mushrooms, but the effects of these factors vary within and across communities. Conducting studies encompassing a broader range of variables is of interest for a better understanding of the human–mushroom relationship.

**Supplementary Information:**

The online version contains supplementary material available at 10.1186/s13002-024-00679-8.

## Introduction

Macromycetes (fungi characterized by the production of sporomes visible to the naked eye commonly known as mushrooms) play a key role in the functioning of most terrestrial ecosystems as mutualists, decomposers of organic matter, and pathogens, but they are also highly relevant in various ethnic groups worldwide as a source of food, medicine, enzymes, and industrial compounds [1–4]. Given their nutritional value (proteins, vitamins, carbohydrates, amino acids, and minerals), some wild mushroom species are used as substitutes for meat and fish in developing countries and are among the most important non-timber forest products sold on the planet, generating approximately US$2 billion annually [3, 5]. Globally, ca. 140,000 mushroom species of importance for ethnic groups have been reported; information obtained from 10 countries revealed the use of 2166 species of wild edible mushrooms, but edible mushrooms are a well-known source of food and income in more than 80 countries. [2, 3].

In Mexico, wild mushrooms are a non-timber forest product highly appreciated due to their ecologic, economic, and cultural value [6]. It has been estimated that more than 450 wild mushroom species are traditionally consumed in Mexico, and at least 20 out of its 62 indigenous groups use this resource [7, 8], making it the second country with the most species of wild mushrooms consumed only after China (ca. 600 species) [9, 10]. The high prevalence of macromycetes and cultural diversity in this country generated vast and complex knowledge about the relationship between humans and wild mushrooms, consolidating ethnomycology as a discipline in charge of studying the human-fungi interrelation, of which Mexico is the pioneer and center of origin [11].

Ethnomycological research allows for a better understanding of the use of mushrooms by ethnic groups and provides a clearer picture of the cosmovision that people have regarding mushroom divinity, ecology, use, and classification [12]. The method most commonly used by ethnomycologists to record information and evaluate culturally important mushroom species is to conduct interviews combined with free lists where people mention the species they know and different issues related to mushrooms [13, 14]. During the first few decades, the data analyses in ethnomycological studies were mainly descriptive and focused on the identification, classification, and traditional use of species [11, 15], but current ethnomycologists complement descriptive methods with quantitative metrics such as the cultural significance of species index, and data are being analyzed using statistical tools [11, 13]. Biological traditional knowledge has intrinsic value and can be assessed without hypotheses and keep scientific rigor using proper methodologies [16]. Thereby, descriptive methods have been suggested to be of great relevance for documenting, organizing, and preserving traditional knowledge (mostly in multi-ethnic countries), and sometimes can generate more complete results than those obtained through quantitative methods [16, 17].

A literature review by Moreno Fuentes and Garibay [18] revealed that 124 ethnomycological studies were published worldwide between 2000 and 2013, and 24% of those studies corresponded to Mexico, the most productive country for ethnomycological research. Most ethnomycological studies in Mexico involve the central and southern regions, and the northern region is the least assessed area. For example, in central Mexico, Ramirez-Carbajal [19] reported that Tlahuica people in the state of Mexico consume 160 mushroom species and have 79 indigenous names and 130 names in Spanish for those species. Servín-Campuzano et al. [20] recorded 16 species used by Purepechas in the state of Michoacan, with an indigenous name for every species. Montoya et al. [21] reported 35 mushroom species used by the Otomi people in the state of Tlaxcala; these species are used as food, medicine, cosmetics, and ornaments. In the state of Mexico, Rodríguez-Muñoz et al. [22] reported 16 species consumed by Nahuatls as food, and their results indicated that women have the highest knowledge on wild mushrooms. In the southern region of Mexico, Shepard et al. [23] observed that Tzotziles and Tzeltales in the state of Chiapas use at least 28 mushrooms and have 70 indigenous names for those species. Ruan-Soto and Ordaz-Velázquez [12] recorded 134 edible and 40 medicinal species used by the Maya people. In the northern region of Mexico, Moreno-Fuentes [24] found that Raramuris in the state of Chihuahua use 16 mushroom species as food and 3 as medicine.

Oaxaca is a Mexican state suggested to be one of the most biodiverse regions in the world, the most biologically and culturally diverse region in Mexico [25, 26], and the most diverse region in this country regarding mushrooms, with 1630 recorded species [27]. Despite Oaxaca being internationally recognized for its vast traditional knowledge about the use of wild mushrooms, there is a lack of ethnomycological studies reporting the wild mushroom species consumed by the different ethnic groups in the region, and studies have been carried out only in 5 (i.e., Zapoteco, Mixe, Mazateco, Mixteco, and Chinanteco) of the 18 indigenous groups of Oaxaca (e.g., [28–30]). The most complete inventory of useful mushrooms in Oaxaca is by Garibay-Orijel et al. [31] in Sierra Norte, and their inventory comprises 159 taxa, including 113 edible species. Additionally, in Sierra Norte, López-García et al. [32] carried out a study in a Chinanteco community and found 36 macrofungal species known by locals, 82% of which were edible, 6% medicinal, 6% toxic, and 6% were ludic mushroom species. In different municipalities of Oaxaca, Martínez-Carrera [33] performed a study on the traditional use, management, and conservation of the Mexican matsutake (*Tricholoma mesoamericanum*) by Zapotecos. In the Mixteca region of Oaxaca, Ruiz-Almenara et al. [29] recorded 138 macromycete taxa, and interviews with local people showed that they consume at least 45 edible species.

Traditional knowledge on wild mushrooms has not been assessed in most of the indigenous groups of Oaxaca, and recording/understanding how this knowledge varies among ethnic groups can be of great relevance for preserving the cultural heritage on wild macromycete species and improving the management and conservation of the fungal resources. This study assessed the traditional knowledge about the use of culturally important wild mushrooms within and across the indigenous groups Chontal, Chatino, and Chinanteco of Oaxaca. Apart from the present study, no ethnomycological studies have been conducted for the Chontal and Chatino groups, and the Chinanteco group has been scarcely considered. These communities are established in different ecosystems and represent distinct cosmologies and cultures, which makes them of great ethnomycological interest. Thus, the present study aimed to (1) determine variations in richness and composition of mushroom species used among the three indigenous groups, (2) unveil the mushroom species that hold greater cultural significance in each studied community, (3) identify differences and similarities in the local nomenclature (indigenous and Spanish) related to wild mushrooms between the studied communities, and (4) determine if the age, level of schooling, and gender affect the distribution of traditional knowledge on wild mushrooms among local people.

## Methods

### Study area

**Fig. 1:**
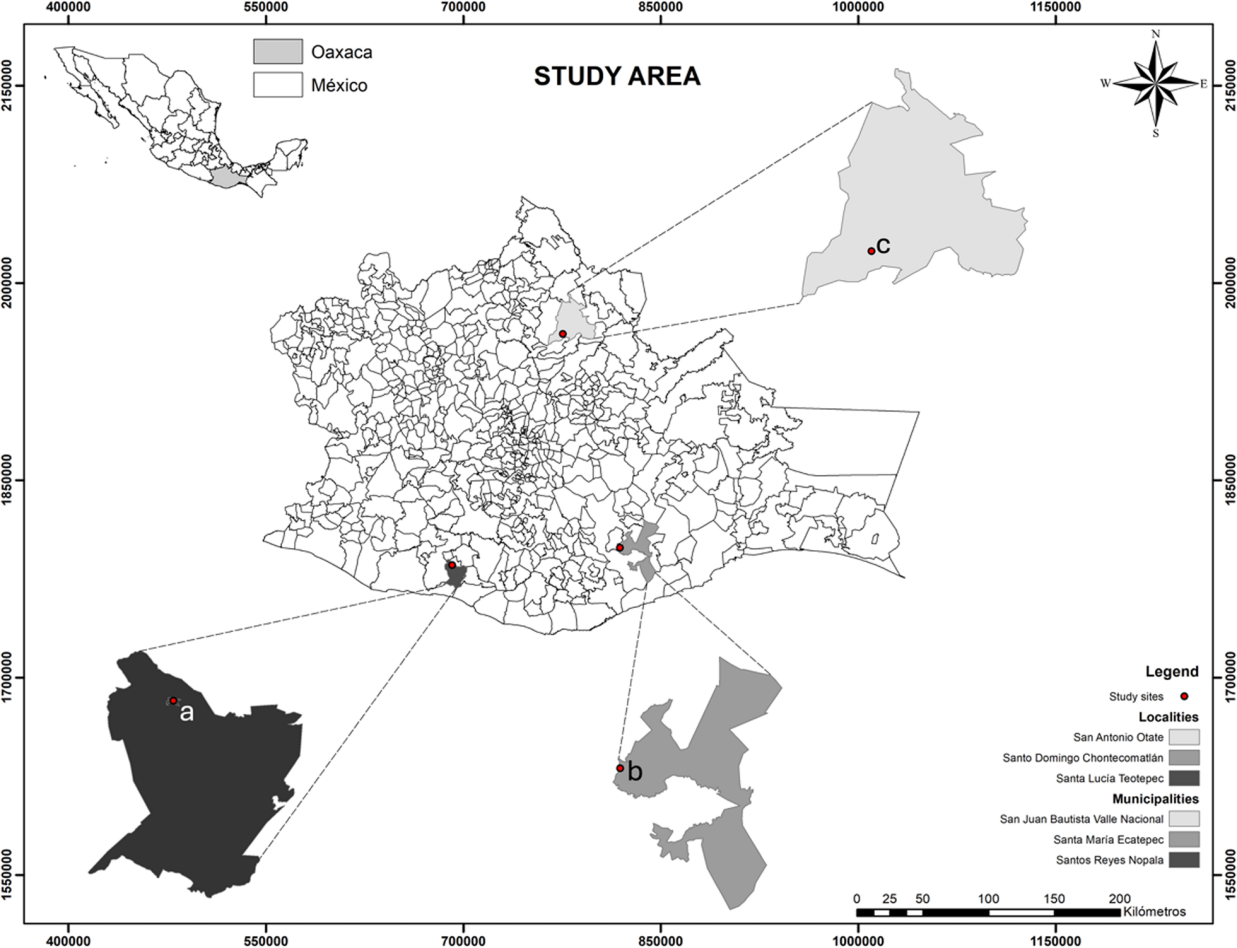
Location of the study sites in different municipalities of Oaxaca, Mexico. The indicated sites are **a** Santa Lucia Teotepec (Chatinos), **b** Santo Domingo Chontecomatlan (Chontales), and **c** San Antonio Otate (Chinantecos). Image by López-García A

The study was conducted within three indigenous communities in the state of Oaxaca, Mexico (Fig. 1).

Santa Lucia Teotepec is part of the municipality of Santos Reyes Nopala located in the region Costa at 16° 8′ 35″ N and 97° 12′ 22″ W, with an elevation of 1172 m. The climate is subtropical dry humid, and its vegetation is the transition from subtropical to temperate forest [34]. The community belongs to the indigenous group Chatino, which comprises 1844 inhabitants, and ca. 80% of them speak the indigenous language [35] (Table 1). Authorization to carry out the study at Santa Lucia Teotepec was given by the municipal agent C. Francisco Sánchez and the interviewed people.

**Table 1:** Environmental and sociodemographic characteristics of the three studied communities in Oaxaca, Mexico

	Santa Lucia Totepec	Santo Domingo Chontecomatlan	San Antonio Oate
Location	16° 8′ 35″ N–97° 12′ 22″ W	16° 15′ 00″ N–96° 01′ 00″ W	17° 71′ 88'' N–96° 40′ 19″ W
Altitude	1172 m	2010 m	336 m
Climate	Subtropical dry humid with summer rainfall, precipitation 1400–1700 mm	Semi-warm subhumid with winter rainfall, precipitation l500–1800 mm	Warm-humid with rainfall throughout the year, precipitation 1200–5000 mm
Vegetation	Pine-oak	Pine-oak	Montane rainforest
Ethnicity	Chatino	Chontal	Chinanteco
Lenguage	Chatino	Chontal	Chinanteco
Religion	Christian	Christian	Christian
Number of inhabitants	1844	388	238
Number of interviewees	62	14	8
Number of interviewees per age range			
7–20	24	3	1
21–40	8	4	3
41–60	20	6	2
61–91	10	1	2
Number of interviewees per gender			
Male	31	7	4
Female	31	7	4
Number of interviewees per occupation			
Housewife	18	6	-
Peasant	16	5	3
Bricklayer	1	–	5
Student	17	2	-
Security guard	1	–	–
Trader	8	1	–
Baker	1	–	–

Santo Domingo Chontecomatlan is part of the municipality of Santa María Ecatepec located in the region Sierra Sur at 16° 15′ 00″ N and 96° 01′ 00″ W, with an elevation of 2010 m. The climate is semi-warm subhumid and characterized by pine-oak vegetation [36]. It belongs to the indigenous group Chontal, comprises 388 inhabitants, and is one of the remaining communities in Oaxaca where people speak the Chontal language [35] (Table 1). Authorization to carry out the study at Santo Domingo Chontecomatlan was given by the municipal agent C. Jorge Mendoza Mejía and the interviewed people.

San Antonio Otate is part of the municipality of San Juan Bautista Valle Nacional located in the region Papaloapan at 17° 71′ 88″' N and 96° 40′ 19″ W, with an elevation of 336 m. Its climate is warm-humid, and the main vegetation is montane rainforest [37]. The community belongs to the indigenous group Chinanteco, which comprises 238 inhabitants, 90% of whom speak the indigenous language [35] (Table 1). Authorization to carry out the study at San Antonio Otate was given by the Supervisory Board and the interviewed people.

### Ethnomycological data obtainment

From April to October 2022, interviews were conducted with a sample size of 10% of the population in every community, which is deemed appropriate because it provides a meaningful representation of the inhabitants, especially when the target populations belong to culturally homogeneous groups. The method proposed by Burrola-Aguilar et al. [38] and Domínguez-Romero et al. [39] was performed to calculate the number of interviewees for each community since it employs a probabilistic cluster sampling, allowing for the random selection of households where any member of a family unit possesses traditional knowledge. Interviews were conducted following guidelines included in the code of ethics of the Latin American Society of Ethnobiology [40]. Authorizations to carry out the study in the Chontal, Chatino, and Chinanteco communities were given verbally by local authorities and interviewed people. For the children, consent was given by their parents.

A total of 84 interviews were conducted: 62 in Santa Lucia Teotepec, 14 in Santo Domingo Chontecomatlan, and 8 in San Antonio Otate. To choose the people being interviewed, houses were randomly selected from the locality maps, and one member of each family was interviewed. The minimum age of the interviewees was 6 years because children in the studied area start participating in field activities at this age. The interviews were aimed at obtaining data about sociocultural issues (e.g., age, economic activity, ethnic group, language, number of family members), what a mushroom is, uses of mushrooms, favorite edible mushrooms, methods to identify edible mushrooms, season of the year to collect mushrooms (phenology), how to cook the mushrooms, medicinal mushrooms, kind of substrata where mushrooms grow, gathering method, the presence of mushrooms in disturbed versus conserved areas, and local names in Spanish and indigenous language for mushroom species and their morphological structures [13, 38, 41, 42] (Additional file 1).

### Macromycete sampling and identification

Macromycete sporomes (mushrooms) were collected only when there was doubt about a species mentioned by an interviewee. A total of 20 species were collected for identification, and voucher specimens were deposited in the mycological collection of the Laboratory of Fungal and Plant Diversity and Evolution-BUAP, Mexico (Additional file 2). The samplings were carried out from May to November 2022 together with people with the highest knowledge on wild mushrooms in each studied locality. The samples were macromorphologically described when fresh [43, 44], and the microscopic descriptions were made from small fragments placed in 5% KOH, Melzer's reagent and 5% Red Congo [45, 46]. Specimens were identified using photos, taxonomic keys, and mycological guides [45, 47–52].

### Cultural importance of wild edible mushrooms

The cultural significance index of wild edible mushrooms (EMCSI) was used to determine the cultural importance of the mushrooms at the studied communities. EMCSI was calculated for every recorded species integrating nine indices, and the information for each index was categorized using the Likert scale [13, 53, 54]:

1. The local nomenclature index (LNI) takes into account the number of words that constitute the indigenous names given by local people to every mushroom species. A value ranging from 0 to 10 was assigned to each species depending on its number of words (Table 2).
2. The mention index (MI) was calculated with the formula [(number of mentions of each species/number of interviewees) × 10].
3. The perceived abundance index (PAI) was categorized from 0 to 10 according to the number of available sporomes for each species during the harvesting season (Table 2).
4. The consumption frequency index (CFI) was obtained based on the number of times a species was consumed by locals during the harvesting season, and the index values range from 0 to 10 (Table 2).
5. The multifunctional food index (MFFI) results from the number of ways the local people cook a species. The index values range from 0 to 10 (Table 2).
6. The consumption preference index (CPI) was obtained from the level of consumption preferences of the species. The interviewees were asked to make a list of the mushroom species they consume in decreasing order from their favorite species to the less preferred species. The index values range from 10 to 1, with 10 being the most preferred species in the list (Table 2).
7. The ability recognition index (ERI) results from the number and types of morphological traits of the mushrooms that people use to identify edible species. The traits were classified on a scale from 0 to 10 (Table 2).
8. The economic index (EI) was calculated based on how people often sell mushrooms and the price at which they sell them. The index values range from 0 to 10 (Table 2).
9. The knowledge transmission index (KTI) takes into account the closeness of the interviewees and the people who transmitted them the knowledge about the use of wild mushrooms. The index values range from 0 to 10 (Table 2).

**Table 2:** Cultural significance sub-indices used to calculate the cultural significance index of wild edible mushrooms

Sub-indices	Answers	Values
LNI	No name	0
	Binomial	2.5
	Trinomial	5.0
	Tetranomial	7.5
	Pentanomial	10
MI	(Amount of mentions/number of interviewees) × 10	
PAI	Null	0
	Rare	2.5
	Medium	5.0
	Common	7.5
	Very common	10
CFI	Never	0
	Not every year	2.5
	Once a year	5.0
	Twice or once a year	7.5
	More than twice a year	10
MFFI	No way of preparation (cook)	0
	One way of preparation	2.5
	Two ways of preparation	5.0
	Three ways of preparation	7.5
	Four ways of preparation	10
CPI	First, second, third, and fourth place	10–7
	Fifth, sixth, and seventh place	6–4
	Eight, ninth, and tenth place	3–1
ERI	They do not know	0
	By its shape, color, and texture	3.3
	By its smell and taste	6.6
	By its color, shape, taste, and place of growing	10
EI	They do not sell or buy	0
	Rarely sell	3.3
	Commonly sell or buy	6.6
	Sell at high prices	10
KTI	An immigrant	2.5
	Local people, not blood parent	5
	Father or mother	7.5
	Three or more generations involved	10

Answers from interviewees were classified and a value was assigned to every classification

*LNI* local nomenclature index, *MI* mention index, *PAI* perceived abundance index, *CFI* consumption frequency index, *MFFI* multifunctional food index, *CPI* consumption preference index, *ERI* edibility recognition index, *EI* economic index, *KTI* knowledge transmission index

The formula used to calculate the cultural significance index of wild edible mushrooms was:

$${\text{EMCSI }} = \, \left( {{\text{LNI}} + {\text{ PAI }} + {\text{ CFI }} + {\text{ MFFI }} + {\text{ CPI }} + {\text{ ERI }} + {\text{ EI }} + {\text{ KTI}}} \right) \, \left( {{\text{MI}}} \right)$$

### Data analyses

Linear regression analyses were performed to determine the relationships between the number of macromycete species known by local people and age and level of schooling (categorized as 1 = no scholar education, 2 = elementary school, 3 = middle school, 4 = high school, 5 = undergraduate, and 6 = higher academic level). Analyses were carried out for all the localities together and for each single locality, both for all the interviewees together and for men and women on their own.

To evaluate differences in the number of known macromycete species between the studied indigenous groups, one-way analysis of variance (ANOVA) was carried out, and Tukey’s HSD tests with 95% confidence level were used to identify pairs of means that differed from each other. To represent on a geometrical plane the distance between the studied groups concerning the composition of the species known by each group, a Non-metric Multidimensional Scaling analysis (NMDS) with 10,000 random starts was performed. The difference between men and women regarding the number of known mushroom species was assessed through two-sample *t* test, both the data of the three indigenous groups together and the data of each group were analyzed. Analyses of covariance (ANCOVAs) were conducted to determine differences between men and women regarding the number of mushroom species they know controlling for the level of schooling and age in both the three study groups together and separately for each group. All the statistical analyses were performed in R Studio [55] (Additional file 3).

## Results

A total of 32 useful macromycete species were recorded in the three studied localities, 30 of them are used as food, and two are used as medicine. People in Santa Lucia Teotepec (Chatinos) consume 23 species, in Santo Domingo Chontecomatl (Chontales) people use 16 species, and 6 species are consumed in San Antonio Otate (Chinantecos). 62% of the recorded species were ectomycorrhizal, 34% saprotrophic, and 3% parasitic (Table 3).

**Table 3:** Wild mushroom species recorded in the studied communities, local indigenous and Spanish names, fungal trophic groups, and local uses

Scientific name	Indigenous names (^1^Chatino, ^2^Chontal, ^3^Chinanteco)	Spanish names (^1^Chatino, ^2^Chontal, ^3^Chinanteco)	Trophic groups	Local uses
*Amanita jacksonii*	^1^Kía kuí nga´a /^2^Jlapilí gunshal	^1^Hongo de San Juan Rojo/^2^Hongo rojo	E	Used as a substitute for meat. Cooked on the griddle with salt, tomato, and onion/fried with onion/in mole, stew, or in Amarillo (a kind of mole traditional of Oaxaca)
*Amanita laurae*	^1^Kía kuí nga´a /^2^Jlapilí gunshal	^1^Hongo de San Juan amarillo/^2^Hongo amarillo	E	Used as a substitute for meat. Cooked on the griddle with salt, tomato, and onion/fried with onion/in mole, stew, or in Amarillo
*Boletus* sp.	^2^Jlapilí chiapaneca	^2^Hongo de chiapaneca	E	Cooked on the griddle with salt, tomato, and onion/cooked on the griddle with salt
*Calvatia* sp.	^2^Jlapilí gogoye	^2^Hongo de calavera	S	Cooked on the griddle with salt/cooked on the griddle with salt and eggs
*Cantharellus cibarius*	^1^Kía kie/^2^Jlapilí kahúa	^1^Hongo de flor/^2^Hongo de calabaza	E	Used as a substitute for meat. Consumed in stew/in mole, or in Amarillo
*Craterellus tubaeformis*	^1^Kía shoo	^1^Hongo de montón	E	Used as a substitute for meat in stew
*Favolus tenuiculus*	^1^Kía jitóo	^1^Hongo de hamaca	S	Used as a substitute for meat. Cooked on the griddle with salt, tomato, and onion/ in stew/cooked on the griddle with salt
*Ganoderma* sp.	^1^Kía shia	^1^Hongo de castilla	S	It is toasted on the griddle, then pulverized and mixed with dough, chili, and Epazote (*Dysphania ambrosioides*)
*Hydnum repandum*	^2^Jlapilí mishto	^2^Hongo de biche	E	Used as a substitute for meat. Consumed in stew or mole/cooked on the griddle with salt, tomato, and onion/cooked on the griddle with salt
*Hygrophorus russula*	^2^Jlapilí gajlene	^2^Hongo de frijol	E	Used as a substitute for meat. Consumed in stew or mole/cooked on the griddle with salt, tomato, and onion/in tamales
*Hypomyces lactiflourum*	^1^Kía jikie lakie/^2^Jlapilí kashi	^1^Hongo cresta de gallo/^2^Hongo de chile	P	Used as a substitute for meat. It is the main ingredient for making salads/cooked on the griddle with salt, tomato, and onion/in stew, or in Amarillo
*Laccaria laccata*	^1^Kía tische/^2^Jlapilí shúlk	^1^Hongo de riata/^2^Hongo de raton	E	Used as a substitute for meat. Cooked on the griddle with salt, tomato, and onion/cooked on the griddle with salt/in stew, or in Amarillo
*Lactarius indigo*	^1^Kía sanandium	^1^Hongo de San Antonio	E	Used as a substitute for meat. Cooked on the griddle with salt, tomato, and onion/cooked on the griddle with salt/in stew
*Lactarius volemus*	^1^Kía squí/^2^Jlapilí fuska-gaja	^1,2^Hongo de leche	E	Used as a substitute for meat. Cooked on the griddle with salt, tomato, and onion/in stew, or in Amarillo
*Lentinus crinitus*	^3^Nat logua quiu	^3^Hongo oreja de tejón	S	Used as a substitute for meat. Cooked on the griddle with salt/in stew
*Neolentinus ponderosus*	^2^Jlapilí góli	^2^Hongo de ocote	S	Used as a substitute for meat. Cooked on the griddle with salt, and onion/in stew, in mole, or in Amarillo
*Pleurotus djamor*	^1^Kía laat /^2^Jlapilí poguimé/^3^Nat majee	^1^Hongo de totomoxtle/^2^Hongo de bobo/ ^3^Hongo de jonote	S	Cooked on the griddle/cooked on the griddle with salt, tomato, and onion/cooked on the griddle with salt, tomato, onion, and Hierba Santa (*Piper auritum*)
*Pseudofistulina radicata*	^1^Kía jikafkhía/^2^Jlapilí ganmamú	^1,2^Hongo de cuachepil	S	Used as a substitute for meat in stew, in mole, or in Amarillo
*Psilocybe* sp.	^1^Kía indiose	^2^Hongo de Dios	S	It is used for healing sessions and consultations in traditional medicine
*Pycnoporus sanguineus*	^3^Nat yöö	^3^Hongo rojo	S	Used as medicine rubbing the sporome on the face to eliminate skin imperfections
*Ramaria flava*	^1^Kía jikaloó /^2^Jlapilí jleúla-keitk	^1^Hongo de corralito/^2^Hongo de cacho de venado	E	Used as a substitute for meat. Cooked on the griddle with salt, and onion/in stew, in mole, or in Amarillo
*Ramaria* sp.	^1^Kía jikaloó /^2^Jlapilí jleúla-keitk	^1^Hongo de corralito/^2^Hongo de cacho de venado	E	Used as a substitute for meat. Cooked on the griddle with salt, and onion/in stew, in mole, or in Amarillo
*Rubroboletus dupainii*	^1^Kía loo	^1^Hongo de molleja	E	Cooked on the griddle with salt, and onion/cooked on the griddle with salt, tomato, onion, and Hierba Santa (*Piper auritum*)
*Russula mexicana*	^1^Kía ishino	^1^Hongo de chile	E	Used as a substitute for meat in stew, in mole, or in Amarillo
*Russula crustosa*	^1^Kía edjee	^1^Hongo de sal	E	Used as a substitute for meat in stew, in mole, or in Amarillo
*Russula* sp. 1	^1^Kía ndad	^1^Hongo de frijol	E	Used as a substitute for meat in stew, in mole, or in Amarillo
*Schizophyllum radiatum*	^1^Kía natho/^3^Nat guo quiic	^1^Hongo de oreja/^3^Hongo mano de lagartija	S	Used as a substitute for meat. Cooked on the griddle with salt/in stew
*Scleroderma* sp.	^1^Kía skuecto	^1^Hongo de huevo	E	Cooked on the griddle with salt/cooked on the griddle with salt and eggs
Tricholomataceae	^3^Nat meec	^3^Hongo de arriera	S	Used as a substitute for meat in mole or in amarillo
*Sparassis crispa*	^3^Nat niiic	^3^Hongo de niebla	E	Used as a substitute for meat in mole, or in stew
*Suillus granulatus*	^1^Kía jaslia/^2^Jlapilí tsepi	^1^Hongo de pan/^2^Hongo de nixtamal	E	Cooked on the griddle with salt, and onion/cooked on the griddle with salt, tomato, onion, and Hierba Santa (*Piper auritum*)
*Xerocomus* sp.	^1^Kía jinóo	^1^Hongo de huarache	E	Cooked on the griddle with salt, and onion

Superscript numbers indicate the community where the indigenous and Spanish names were recorded

*E* ectomycorrhizal, *S* saprobic, *P* parasite

The oldest people in the studied communities have the greatest knowledge on the traditional use of wild mushrooms and are responsible for transmitting this knowledge to younger people. Men collect mushrooms growing in forest areas far from communities, and women and children collect in surrounding lands.

Information from the interviews in the three localities indicated that the harvesting season begins in June and finishes in October. People suggest that most macromycete species produce sporomes within a specific time span during the rainy season, but production may be influenced by environmental variations. They can harvest up to 5 kg of mushrooms in 1 day and classify the species based on the kind of substrata where they grow.

To preserve this non-timber forest product, mushroom pickers apply different traditional methods. To collect ectomycorrhizal species, they cut the stipe to leave the basal part of the stipe in the ground. Additionally, people slightly tap the pileus to help release the spores. In the case of xylophagous fungi, local gatherers avoid removing or disturbing the logs and branches where useful mushrooms have been collected.

People from the three studied groups are aware of the negative effect that deforestation can generate on the fructification rates of species such as *Cantharellus cibarius*, *Amanita* spp. and *Russula* spp., so they conserve harvesting areas by avoiding timber extraction.

### Local nomenclature

Every studied ethnic group gives local indigenous and Spanish names to the different macromycete species they know (Table 3). The indigenous names are composed of the word that every community uses for “mushroom”, together with one or two epithets that refer to the morphologic and/or ecological features of the species.

The indigenous word that Chatinos use for “mushroom” is *Kía* and is used at the beginning of a species name. For example, they consume a *Russula* species named *Kía edjee* in the local language, which means “mushroom of salt” because the species has scales on the pileus, and the name in Chatino for scales is salt (*edjee*). The macrofungal species *Pseudofistulina radicata* is named *Kía jikafkhía* because it grows on a woody plant of the genus *Diphysa* named *jikafkhía* by locals. Some names are given out of the shape of the mushrooms, for example, the name for *Hypomyces lactifluorum* is *Kía jikie lakie*, which means “cockscomb mushroom”. Due to its shape and color, the species *Rubroboletus dupainii* is named *Kía loo*, which means “gizzard mushroom” (Fig. 3a). In the Chatino community, the names of the edible *Amanita* species are trinomials. The names are *Kía kuí nga´a* (red San Juan mushroom) and *Kía kuii gsi* (yellow San Juan mushroom) due to their color and because they grow in June, the month of San Juan.

Correspondingly, indigenous names in the Chontal group are given based on the morphologic traits of the species. For example, the color of the species *Cantharellus cibarius* is yellow, similar to the color of the pumpkin flower, thus, the local name is *Jlapilí kahúa*, which means “pumpkin mushroom”. The species *Lactarius volemus* releases latex (white liquid resembling milk) when the sporome is cut, and its indigenous name is *Jlapilí fuska-gaja*, which means “milk mushroom”. *Hydnum repandum* is a species with teeth on the hymenium, similar to the cats’ tongue, and its local name is *Jlapilí mishto*, which means “cat mushroom”.

Chinantecos use binomial and trinomial indigenous names for the mushrooms, and both the morphologic and ecological traits of the species are used to name them. For example, the local name for *Pleurotus djamor* is *Nat majee*, which means “jonote mushroom”. Jonote is a tree species (*Heliocarpus appendiculatus*) that has been observed to be associated with the distribution of *P. djamor*. The macromycete *Schizophyllum radiatum* is named *Nat logua quiic*, which means “lizard hand mushroom” because the sporome resembles a scaly lizard hand. In the three studied indigenous groups, there is a presence on the use of ethnotaxas (a single indigenous name including several macromycete species that are morphologically alike).

### Mushroom morphology

**Fig. 2:**
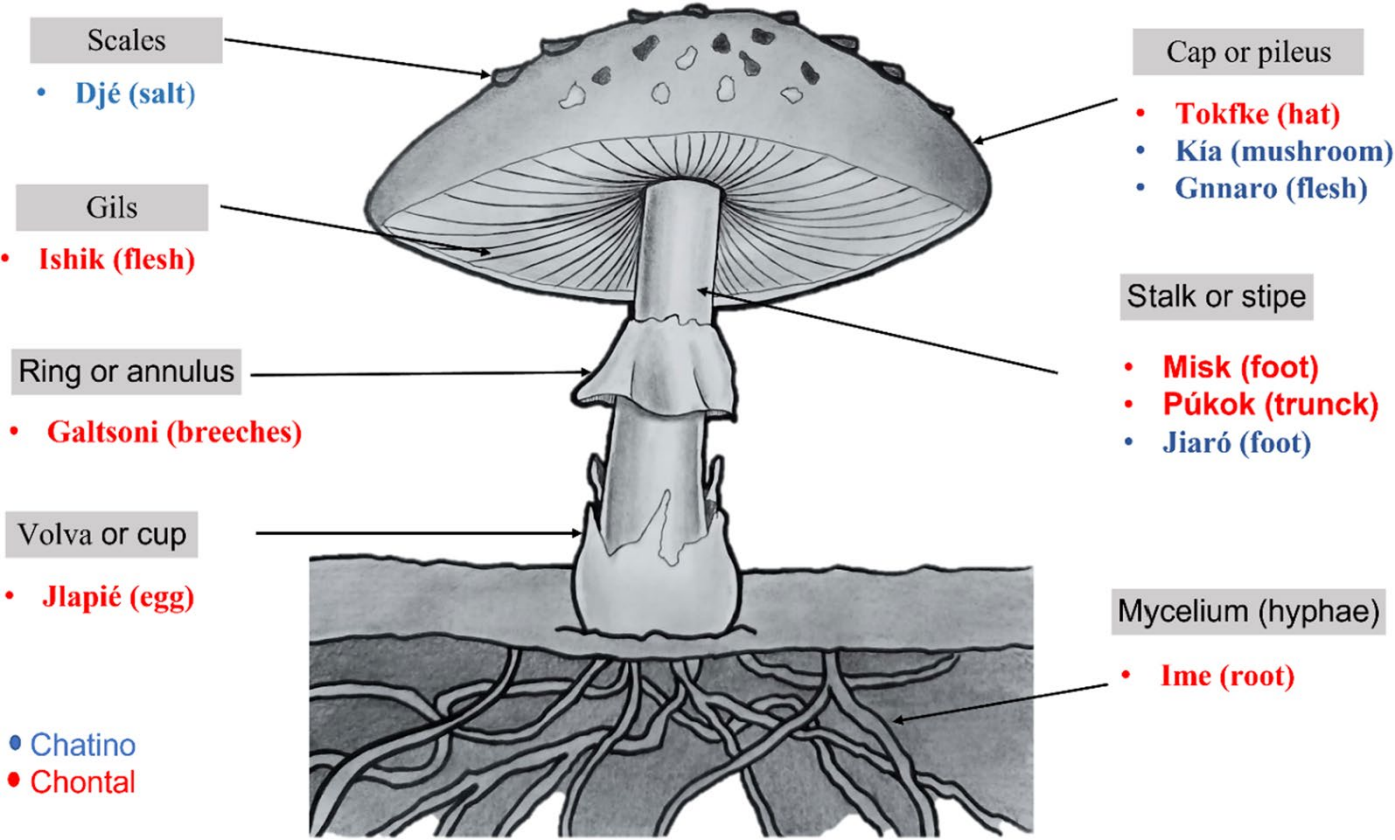
Local indigenous names of the morphological structures of an agaricoid mushroom recorded in the Chatino and Chontal communities

Naming the different morphological structures of mushroom sporomes seems irrelevant for Chinantecos and Chatinos. Chinantecos do not have any name for the parts of the sporome. Chatinos have indigenous names for only three parts; the stipe is named *Jiaró* (paw), the pileus name is *Kia* or *Gnnaro* (skull or meat), and the name for the scales is *Edjee* (salt). The latter is the most important structure because it is useful for distinguishing edible and toxic species (Fig. 2).

Differentiating the parts of a sporome is important for Chontales. They have indigenous names for seven structures and use homologies of common things around to name them. For example, the name for the pileus is *Tokfke* (hat), the hymenium is named *Ishik* (meat), the stipe is *Misk* (paw) or *Pútok* (tronco), the mycelium is *Ime* (root), and the volva is *Jlapié* (egg). For Chontales, the annulus is the key structure for determining whether a species is edible because the mushrooms they consume have a large yellow annulus, and the local name for this structure is *Galtsoni* (briefs) (Fig. 2).

### Ethnoecological knowledge about wild mushrooms

Interviewees from the three studied indigenous groups showed to be aware that plants and mushrooms are distinct types of organisms, but they need each other to grow in forests. They mentioned that wild mushrooms play several roles in ecosystem functioning, mainly in processes related to soil fertility. Moreover, people use diverse criteria to classify mushrooms according to their ecological role, and the most used classification is the type of substrate on which they grow.

Regarding mushroom phenology, people in the three indigenous groups mentioned that there are long-availability species, such as *Schizophyllum commune* and *Lentinus crinitus,* that grow throughout the complete rainy season, and agricultural activities like the clearcut-and-burn method enhance sporome production in some species. Moreover, they have observed that mushroom production improves in June and September with increasing precipitation.

However, 80% of the interviewees said that the use of agricultural chemical agents, the immoderate felling of trees, and changes in precipitation patterns have led to a decline in sporome production.

To preserve fungal resources, people avoid cutting trees in old forest stands because these areas produce greater amounts of mushrooms, they leave pieces of stipe on the ground to ensure future harvests, and they do not collect mature sporomes to allow the release of seeds (spores).

### Medicinal mushrooms

The use of mushrooms in traditional medicine was found only in Santa Lucia Teotepec (Chatinos) and San Antonio Otate (Chinantecos). Chatinos use *Psilocybe* species in healing and prediction ceremonies. The ceremonies are carried out at night by someone with experience, and the mushrooms are smoked with copal before they are consumed. Chatinos consume two or three sporomes in ceremonies. Once the mushrooms take effect, people hear a voice in their head and ask that voice for answers about how to cure a sickness or solve a problem. Chinantecos use *Pycnoporus sanguineus* due to its properties to remove skin blemishes by applying the sporome on their face to cover the skin with spores, which are the curative component of the mushroom.

### Cultural significance of wild edible mushrooms for the Chatino group

**Fig. 3:**
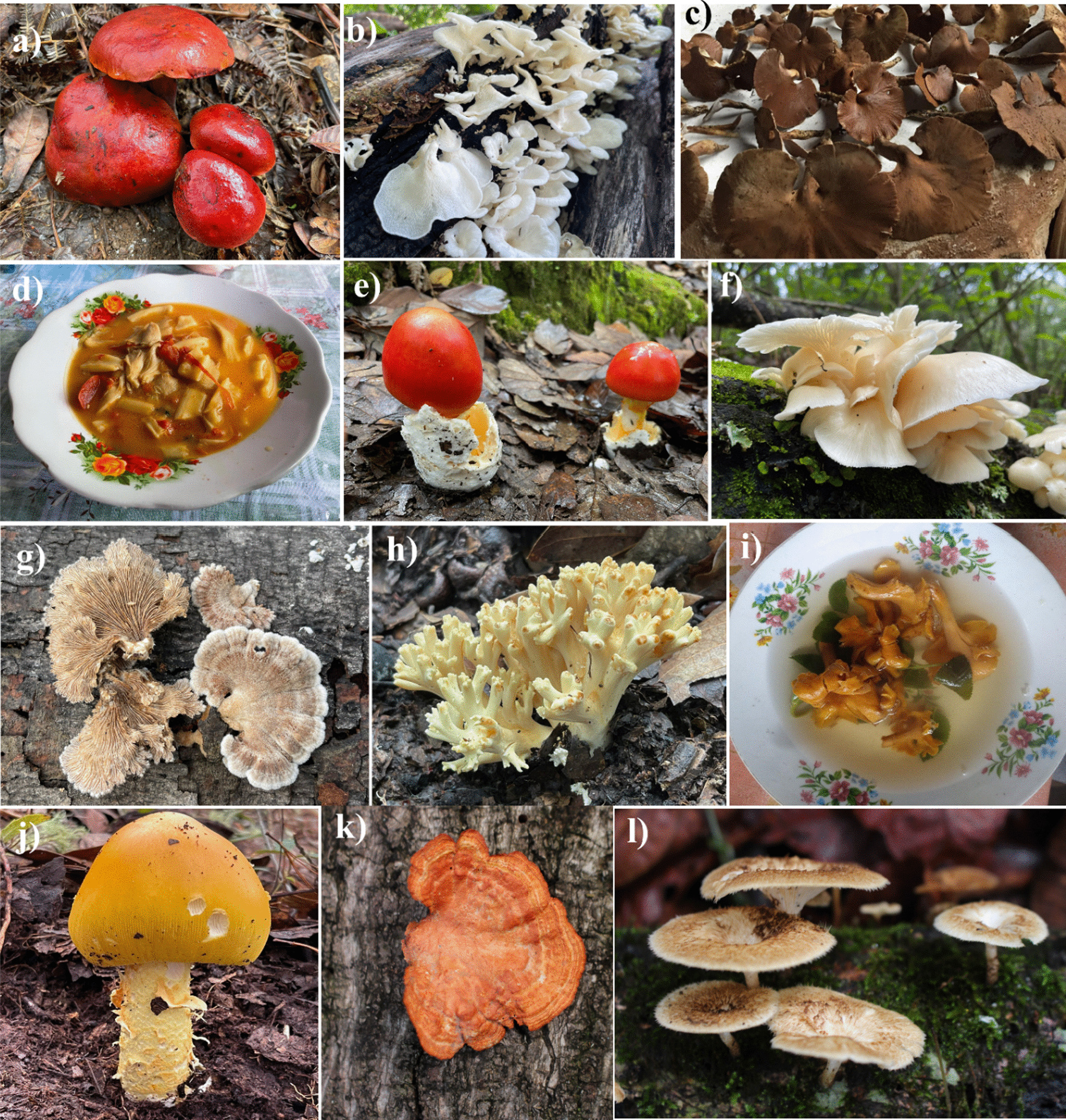
Wild mushrooms of biocultural importance in the Chinanteco, Chontal, and Chatino communities of Oaxaca. The images show **a** *Rubroboletus dupainii,* **b** *Favolus tenuiculus*, **c** *Pseudofistulina radicata*, **d** stew made with species of the genus *Amanita,* **e** *Amanita jacksonii*, **f** *Pleurotus djamor*, **g** *Schizophyllum radicatum*, **h** *Ramaria* sp., **i** stew made of *Cantharellus cibarius*, **j** *Amanita laurae*, **k** *Pycnoporus sanguineus*, and **l** *Lentinus crinitus*

The five macromycete species with the highest cultural significance index for wild edible mushrooms in the Chatino community were “salt mushroom” (*Russula* sp.1), “bean mushroom” (*Russula* sp.2), “totomoxtle mushroom” (*Pleurotus djamor*), “red San Juan mushroom” (*Amanita jacksonii*) (Fig. 3e), and “cuachepil mushroom” (*Pseudofistulina radicata*) (Table 4).

**Table 4:** Cultural importance of the wild edible mushroom species recorded in the Chatino group

Species	MI	LNI	CFI	CPI	PAI	EI	ERI	ITC	MFFI	EMCSI
*Russula crustosa*	7.74	2.50	8.44	9.19	7.92	2.41	3.79	8.65	5.57	375.15
*Russula* sp.	5.32	2.50	8.03	8.48	7.42	0.60	3.71	8.56	5.45	238.24
*Pleurotus djamor*	4.52	2.50	7.90	9.71	7.66	1.38	5.89	8.71	5.24	221.30
*Amanita jacksonii*	4.68	5.00	6.64	8.76	5.34	1.37	5.15	8.36	5.17	214.21
*Pseudofistulina radicata*	4.35	2.50	7.59	9.22	7.59	1.83	6.03	8.61	5.46	212.71
*Cantharellus cibarius*	4.52	2.50	7.77	9.25	7.32	1.18	4.98	8.39	4.82	208.68
*Russula mexicana*	3.87	2.50	7.81	8.67	7.19	1.10	4.00	8.65	5.83	177.06
*Ganoderma* sp.	3.39	2.50	6.90	9.48	6.79	1.26	5.37	8.81	5.95	159.39
*Lactarius volemus*	2.90	2.50	7.50	8.28	6.39	0.37	3.48	8.89	5.56	124.73
*Schizophyllum radiatum*	2.58	2.50	8.44	8.94	7.66	0.00	3.93	8.75	5.78	118.68
*Amanita laurae*	2.42	5.00	8.00	9.20	6.00	0.67	4.64	8.67	6.33	117.35
*Craterellus tubaeformis*	2.58	2.50	7.19	8.44	6.56	0.41	4.98	8.28	5.31	112.69
*Rubroboletus dupainii*	2.42	2.50	7.17	8.60	6.00	0.88	3.97	8.67	4.83	103.10
*Scleroderma* sp.	2.10	2.50	7.31	8.15	6.73	0.51	4.85	8.85	5.58	93.24
*Lactarius indigo*	1.13	2.50	8.57	8.71	6.79	0.00	4.73	8.57	5.71	51.47
*Suillus granulatus*	0.81	2.50	6.50	6.80	4.00	1.20	4.64	8.50	3.50	30.35
*Ramaria* spp.	0.48	2.50	9.17	7.67	8.33	0.00	3.30	9.17	7.50	23.05
*Xerocomus* sp.	0.48	2.50	7.50	7.33	5.83	0.00	3.30	9.17	4.17	19.26
*Laccaria laccata*	0.32	2.50	7.50	8.50	6.25	1.65	3.30	7.50	2.50	12.81
*Hypomyces lactiflourum*	0.16	5.00	10.00	10.00	10.00	0.00	3.30	10.00	10.00	9.40
*Favolus tenuiculus*	0.16	2.50	7.50	8.00	10.00	0.00	10.00	10.00	7.50	8.95

*MI* mention index, *LNI* local nomenclature index, *CFI* consumption frequency index, *CPI* consumption preference index, *PAI* perceived abundance index, *EI* economic index, *ERI* edibility recognition index, *KTI* knowledge transmission index, *MFFI* multifunctional food index, *EMCSI* cultural significance index of wild edible mushrooms

Except for *Amanita jacksonii* (Fig. 3e), *A*. *laurae* (Fig. 3j) and *Hypomyces lactifluorum* (index values = 5), the local nomenclature index indicated that the names assigned to the mushroom species are not complex because they are composed mainly of morphological traits (Table 4).

The perceived abundance index suggested that Chatinos perceive *Hypomyces lactifluorum*, *Favolus tenuiculus* and *Ramaria* spp. as the most abundant species (index values > 8), whereas *Suillus granulatus*, *Xerocomus* sp. and *Amanita jacksonii* are the least abundant species (index values < 6).

The consumption frequency index showed that all the species are eaten at least once a year, with *Hypomyces lactifluorum* and *Ramaria* spp. being the most frequently consumed species (index values > 6).

Regarding the different ways to cook wild mushrooms, the multifunctional food index indicated that all the recorded species, except *Laccaria laccata* (index value = 2.5), are cooked in at least two ways (index values ≥ 7.5).

The consumption preference index indicated that all mushroom species are prized due to their good flavor. *Hypomyces lactiflourum*, *Pleurotus djamor*, *Ganoderma* sp. and *Cantharellus cibarius* were the most preferred by Chatinos (index values > 7), whereas *Xerocomus* sp. and *Ramaria* spp. were the least preferred.

The low values obtained with the edibility recognition index suggested that identifying edible mushrooms is easy for Chatinos because identification is based mainly on mushroom morphology. In the case of *Favolus tenuiculus,* identification is more complicated (index value = 10) because it includes the growing habitat, flavor, and smell.

Regarding wild mushroom marketing in the community, the economic index indicated that *Lactarius indigo*, *Schizophyllum radiatum*, *Ramaria* spp., *Favolus tenuiculus,* and *Hypomyces lactiflourum* do not represent a sale item (index values = 0). *Russula crustosa* was the species with the highest value (index value = 2.4), and its price can reach approx. USD$17.5 per kilogram.

The knowledge transmission index indicated that the knowledge on wild edible mushrooms within the Chatino community goes back to at least one generation (index values > 7.5).

### Cultural significance of wild edible mushrooms for the Chontal group

The five macromycete species with the highest cultural significance index for wild edible mushrooms in the Chatino community were *Laccaria laccata*, *Amanita laurae*, *Cantharellus cibarius*, *Lactarius volemus* and *Hypomyces lactifluorum* (index values > 350). Flavor, multifunctionality, and perceived abundance were the main factors influencing the preference of these species for Chontales (Table 5).

**Table 5:** Cultural importance of wild edible mushrooms in the Chontal group

Species	MI	LNI	CFI	CPI	PAI	EI	ERI	KTI	MFFI	EMCSI
*Laccaria laccata*	9.28	2.50	9.03	9.30	9.42	0.50	3.30	8.07	6.34	450.35
*Amanita laurae*	9.28	2.50	9.61	9.92	7.88	0.00	3.30	8.07	7.11	449.57
*Cantharellus cibarius*	8.57	2.50	9.16	9.50	7.70	0.00	3.30	7.91	6.45	399.00
*Lactarius volemus*	7.85	2.50	9.37	10.63	8.54	1.10	3.30	7.91	6.45	391.50
*Hypomyces lactiflourum*	7.85	2.50	8.63	9.54	7.95	0.60	3.30	7.95	6.81	371.71
*Amanita jacksonii*	7.14	2.50	9.50	9.90	7.00	0.00	3.30	8.00	8.00	344.28
*Ramaria* sp.	4.28	2.50	9.16	9.33	9.16	0.00	3.30	7.91	6.25	204.14
*Neolentinus ponderosus*	3.57	2.50	6.00	10.00	5.00	0.00	3.30	8.00	8.50	154.64
*Pleurotus djamor*	2.14	2.50	7.50	6.00	6.25	0.00	3.30	8.75	8.75	92.25
*Hydnum repandum*	1.42	2.50	10.00	10.00	7.50	0.00	3.30	7.50	6.25	67.21
*Suillus granulatus*	1.42	2.50	8.75	9.00	7.50	0.00	3.30	7.50	5.00	62.21
*Calvatia* sp.	1.42	2.50	10.00	10.00	2.50	0.00	3.30	7.50	7.50	61.85
*Boletus* sp.	0.71	2.50	10.00	10.00	10.00	0.00	3.30	7.50	7.50	36.28
*Pseudofistulina radicata*	0.71	2.50	10.00	10.00	10.00	0.00	3.30	7.50	7.50	36.28
*Hygrophorus russula*	0.71	2.50	5.00	10.00	10.00	0.00	3.30	7.50	7.50	32.71

*MI* mention index, *LNI* local nomenclature index, *CFI* consumption frequency index, *CPI* consumption preference index, *PAI* perceived abundance index, *EI* economic index, *ERI* edibility recognition index, *KTI* knowledge transmission index, *MFFI* multifunctional food index, *EMCSI* cultural significance index of wild edible mushrooms

The local nomenclature index suggested that all the local names given to the mushroom species are comprised of two terms (index values = 2.5), and the myconomies assigned to the species are based on morphological traits.

*Boletus* sp., *Pseudofistulina radicata,* and *Hygrophorus russula* are perceived as the most abundant mushroom species and as the easiest to find in the forest based on the perceived abundance index (values = 10). The rarest species was *Calvatia* sp. (index value = 2.5). The remaining species had values indicating they are not rare and easily found (Table 5).

The values calculated with the consumption frequency index indicated that most species are consumed at least twice a year (index values ≥ 7.5), but the species *Calvatia* sp. *Boletus* sp., *Hydnum repandum* and *Pseudofistulina radicata* are consumed more than four times in the year (index values = 10). *Hygrophorus russula* and *Neolentinus ponderosus* are consumed once a year (values = 5 and 6, respectively) (Table 5).

The multifunctional food index indicated that all the recorded species in the Chontal community are cooked in at least two ways (index values ≥ 5).

Except for *Pleurotus djamor*, the values calculated with the consumption preference index suggested that Chontales appreciate most of the mushroom species due to their good flavor (index values ≥ 9). People in the community do not completely appreciate the flavor of *P. dejamor* (index value = 6) (Table 5).

The values obtained with the edibility recognition index for all the wild edible mushroom species known by Chontales indicated that people collect those mushrooms with confidence that all of them are edible species (index values 3.3).

The economic index showed that only *Lactarius volemus*, *Laccaria laccata* and *Hypomyces lactifluorum* are commercialized by Chontales (index values = 1.1, 0.5 and 0.6, respectively). The price of these mushroom species can vary from approx. USD$5.8 to $8.8 per kilogram depending on their abundance along the rainy season.

The knowledge transmission index values indicated that the knowledge on wild edible mushrooms within the Chontal community goes back to at least one generation (index values ≥ 7.5).

### Cultural significance of wild edible mushrooms for the Chinanteco group

In the Chinanteco community, people consume only five mushroom species. *Pleurotus djamor* (Fig. 3f) is the most important species in this community, with 513.62 in the cultural significance index for wild edible mushrooms, whereas *Sparassis crispa* is the least important with an index value = 45.37 (Table 6).

**Table 6:** Cultural importance of wild edible mushrooms in the Chinanteco group

Species	MI	LNI	CFI	CPI	PAI	EI	ERI	KTI	MFFI	EMCSI
*Pleurotus djamor*	10.00	5.00	7.18	9.37	6.25	6.60	4.13	8.12	4.68	513.62
*Lentinus crinitus*	3.75	7.50	5.83	8.33	6.66	0.00	5.53	7.50	4.16	170.75
Tricholomataceae	2.50	5.00	3.75	9.50	2.50	0.00	6.65	7.50	3.75	96.62
*Schizophyllum radiatum*	3.75	7.50	6.66	9.00	5.83	0.00	3.30	8.33	3.33	164.87
*Sparassis crispa*	1.25	5.00	2.50	8.00	2.50	0.00	3.30	10.00	5.00	45.37

*MI* mention index, *LNI* local nomenclature index, *CFI* consumption frequency index, *CPI* consumption preference index, *PAI* perceived abundance index, *EI* economic index, *ERI* edibility recognition index, *KTI* knowledge transmission index, *MFFI* multifunctional food index, *EMCSI* cultural significance index of wild edible mushrooms

All the mushrooms consumed in this community have indigenous names. The local nomenclature index showed that the local names for *Lentinus crinitus* and *Schizophyllum radiatum* are comprised of three terms (index values = 7.5), and the names for the rest of the mushrooms are comprised of two terms (index values = 5) (Table 6).

The perceived abundance index indicated that *Pleurotus djamor* and *Lentinus crinitus* are the most abundant species, however, the classification used to calculate the index suggested that those species are in a medium level of abundance (index values < 7.5*). Sparassis crispa* was the rarest species according to the Chinanteco people (index value = 2.5).

The consumption frequency index suggested that *Sparassis crispa* and an unidentified Tricholomataceae species are not consumed every year (index values < 3.75) and the other mushrooms are consumed at least once a year (values > 5) (Table 6).

The multifunctional food index indicated that the mushroom species consumed by locals are cooked in at least one way (index values > 3).

The values obtained with the consumption preference index indicated that all the species are consumed due to their good flavor (index values ≥ 8).

*Pleurotus djamor* is the only species commercialized in the Chinanteco community and obtained a high economic index (index value = 6.6) (Table 6). The price of this mushroom can reach more than USD$11.5 per kilogram.

The knowledge transmission index indicated that the knowledge on wild edible mushrooms in the Chinanteco community goes back to at least one generation (index values ≥ 7.5). *Sparassis crispa* is not a species traditionally consumed by Chinantecos, it was introduced to the community by external people, however, it is the only species known from at least two generations back (index value = 10).

Distribution of traditional knowledge about wild edible mushrooms in the Chatino, Chontal, and Chinanteco groups.

**Fig. 4:**
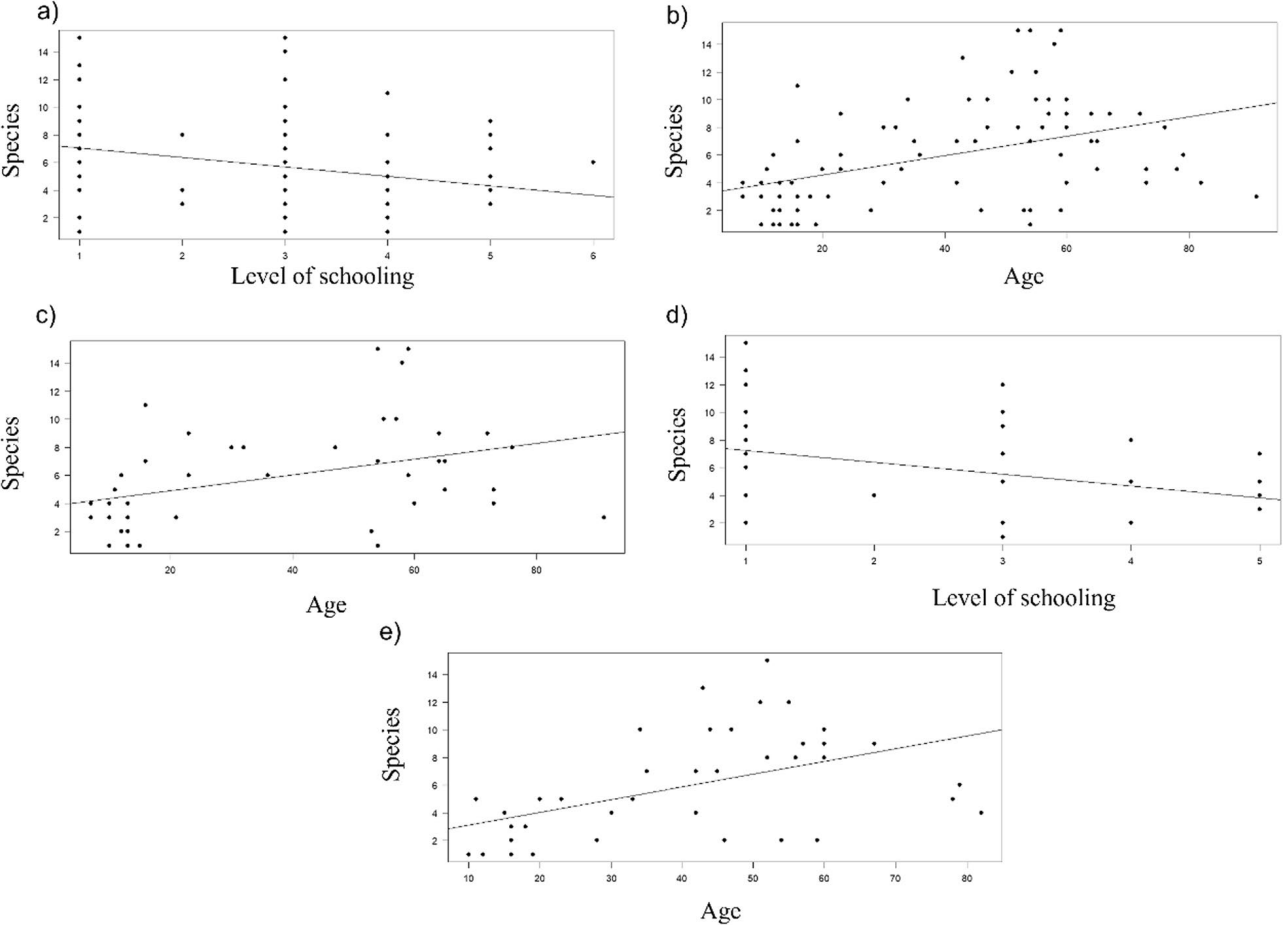
Linear regression analysis. The number of known mushroom species recorded in the three studied communities relates to **a** the level of schooling of men and women, **b** the age of men and women, **c** the age of men, **d** the level of schooling of women, and **e** the age of women

The linear regression analyses indicated that the number of wild edible mushrooms identified by the interviewed people in the three studied groups was negatively related to their level of schooling (*r*^2^ = 0.07, *F* = 5.85, *p* = 0.017; Fig. 4a) and positively related to their age (*r*^2^ = 0.19, *F* = 19.2, *p* < 0.0001; Fig. 4b).

The number of mushroom species known by all the men in the three studied groups was not significantly related to their level of schooling (*p* > 0.5) but was positively related to their age (*r*^2^ = 0.15, *F* = 6.7, *p* = 0.011; Fig. 4c). However, the number of species known by the women was negatively related to their level of schooling (*r*^2^ = 0.12, *F* = 5.4, *p* = 0.02; Fig. 4d), and positively related to their age (*r*^2^ = 0.25, *F* = 13.6, *p* < 0.0001; Fig. 4e).

**Fig. 5:**
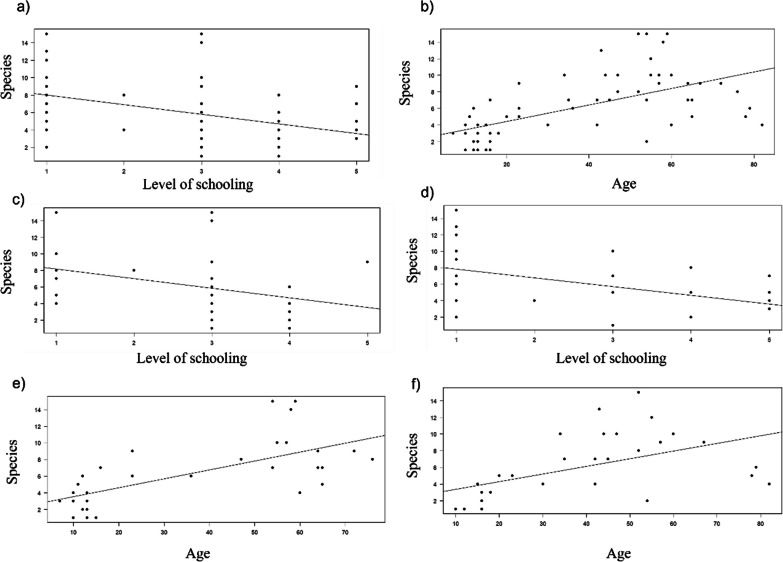
Linear regression analysis. The number of known mushroom species recorded in the Chatino community relates to **a** the level of schooling of men and women, **b** the age of men and women, **c** the level of schooling of the men, **d** the level of schooling of women, **e** the age of men, and **f** the age of women

In the Chatino group, the knowledge about mushroom species obtained from all the interviewees was negatively related to their level of schooling (*r*^2^ = 0.17, *F* = 11.94, *p* = 0.001; Fig. 5a), and positively related to their age (*r*^2^ = 0.35, *F* = 33.1, *p* < 0.0001; Fig. 5b). Similarly, the number of species known by both men and women in the Chatino community was negatively related to their level of schooling (*r*^2^ = 0.13, *F* = 4.3, *p* = 0.04; *r*^2^ = 0.2, *F* = 7.41, *p* = 0.01, respectively; Fig. 5c, d), and positively related to their age (*r*^2^ = 0.45, *F* = 24.6, *p* < 0.0001; *r*^2^ = 0.27, *F* = 10.63, *p* = 0.002, respectively; Fig. 5e, f). The number of wild edible mushrooms known by the people from the Chontal and Chinanteco groups was not significantly related to their level of schooling or age (*p* > 0.5).

**Fig. 6:**
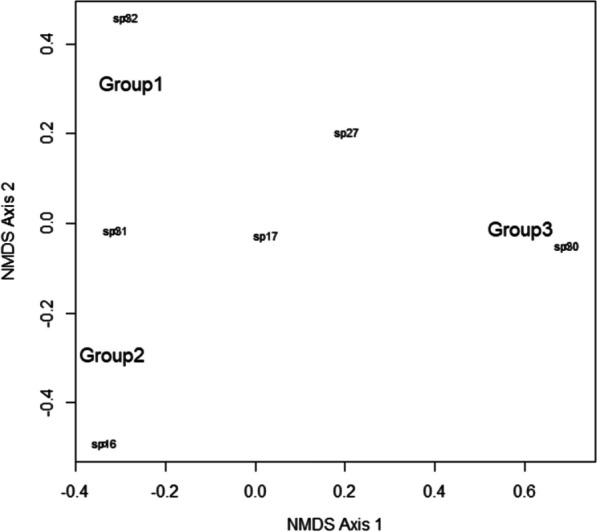
Non-metric multidimensional scaling analysis. Group 1 (Chatinos), Group 2 (Chontales), and Group 3 (Chinantecos). The distance between groups represents the similarity in the species composition they consume

The ANOVA indicated highly significant differences between the Chatino, Chontal, and Chinanteco groups regarding the number of mushrooms they know and use (*F* = 10.9, *p* < 0.0001). Tukey’s test showed that the main difference is between the Chatino and Chinanteco groups (*p* < 0.0001), followed by the Chatino and Chontales groups (*p* = 0.02). Similarly, the NMDS indicated that the Chinanteco group is clearly separated from the Chatino and Chontal groups along Axis 1 due to differences in the composition of the species they use (Fig. 6).

**Fig. 7:**
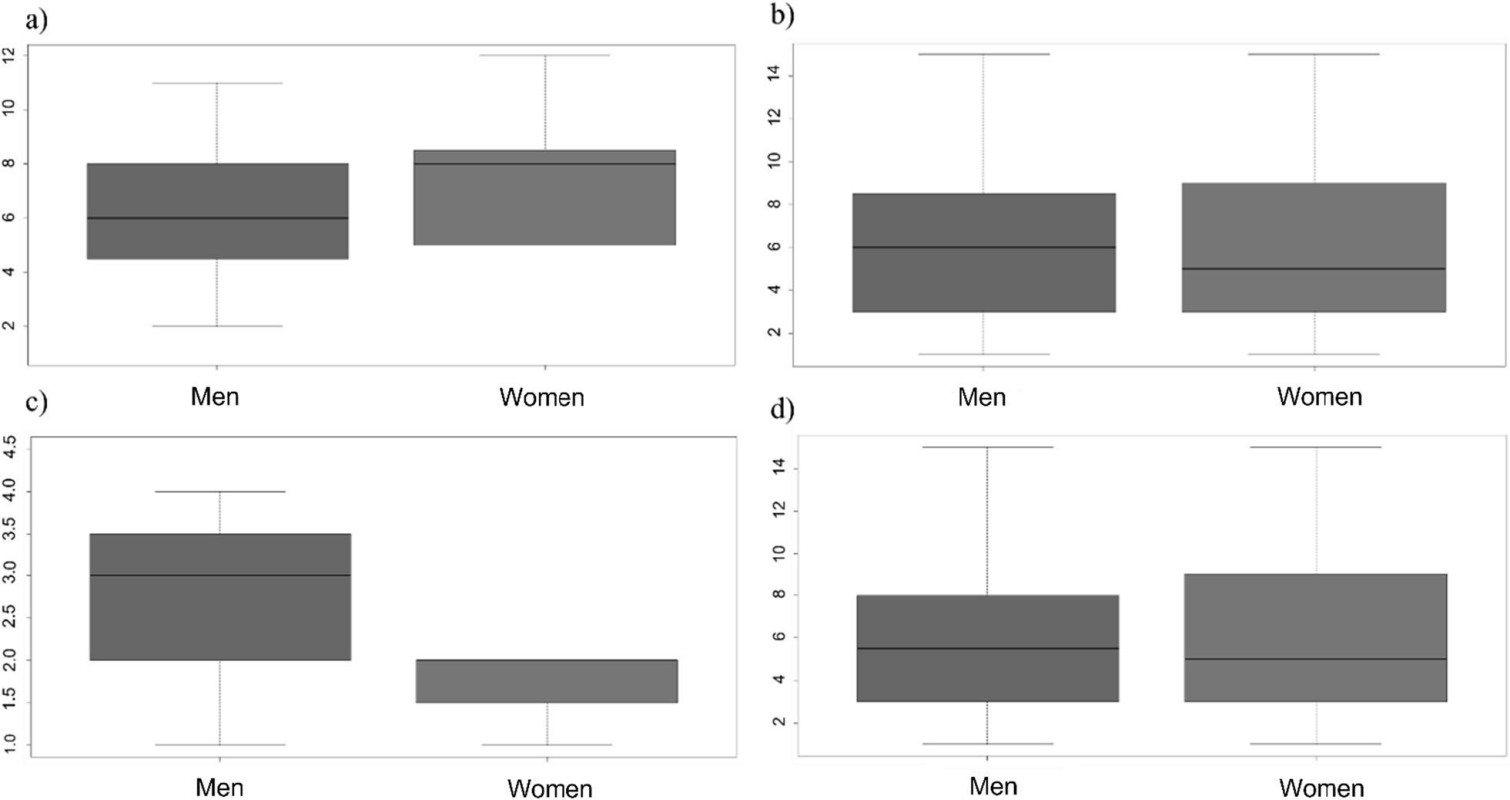
Student’s *t* test. Differences in the number of mushroom species known by **a** men and women in the three communities, **b** men and women in the Chatino group, **c** men and women in the Chontal group, and **d** men and women in the Chinanteco group. There are no significant differences between men and women regarding the number of useful mushroom species they know

The *t* test indicated that there are no significant differences between men and women regarding the number of useful mushroom species they know, the results were analyzed both for the three study groups together and for each group individually (*p* > 0.05; Fig. 7a–d). However, the ANCOVAs controlling for level of schooling and age in the three groups together and in the Chatino group showed that there is a significant difference between men and women in terms of the number of mushroom species they know after controlling for age (*F* = 18.86, *p* < 0.0001; *F* = 32.32, *p* < 0.0001, respectively); however, the difference was not significant in the Chinanteco and Chontal groups (*p* > 0.05).

## Discussion

The different kinds of knowledge arising from the human–mushroom relationship have been acquired, accumulated, and specialized over the years. These broad forms of knowledge are complex and differ among ethnic groups in Mexico and worldwide, as shown by the results obtained in the present study [56–58]. It has been suggested that mushroom species used by human groups inhabiting the same ecological zone can be similar but used in distinct ways [59], corresponding to the observed between the Chontal and Chatino communities. The appropriation of wild mushrooms by ethnic groups has led to the diversification of their use and, over time, these organisms have been incorporated as an element of great importance in their cultural core.

Mexico stands out as the region where most of the ethnomycological research is carried out, encompassing about a quarter of all the ethnomycological studies published worldwide [14, 18]. The traditional knowledge on wild mushrooms has been evaluated in several ethnic groups along the country, and the findings have allowed a better understanding of the cognitive systems generated through the interaction of human groups and fungi. Ethnomycological studies are mainly descriptive and focused on recording the useful mushroom species, harvesting methods, mushroom-related nomenclature, and ethnoecological knowledge in different ethnic groups (Table 7), but studies analyzing the variation of traditional knowledge within and across groups are scarce. In addition to recording and preserving valuable ethnomycological information, this study performed quantitative analyses to explore how traditional knowledge distributes within and across the indigenous communities, and factors influencing the distribution. Addressing the traditional knowledge under different approaches can be instrumental in preserving and valuing cultural diversity, as well as fostering sustainable development.

**Table 7:** Ethnomycological studies in Mexico and their main findings related to the traditional knowledge on the use of wild mushrooms in different indigenous groups

References	Indigenous groups	Main findings
Montoya et al. [21]	Otomí	The use of 35 mushroom species in the state of Tlaxcala was reported. They are used as food, medicine, cosmetics, and ornament
López-García et al. [60]	Chinanteco	For the first time, the ethnomycology of the Chinantec group was documented. A total of 36 species were reported; 31 used as food, 3 as medicine, and 2 as recreative to write or draw on them
Garibay-Orijel et al. [13]	Zapoteco	The Cultural Significance Index of Wild Edible Mushrooms was shown for the first time in a published article. The significance value of 37 species of mushrooms was analyzed
Hernández-Santiago et al. [61]	Mixteco	The use, nomenclature, classification, ecology, and gastronomy of 26 edible species were reported. Additionally, they reported 6 species used for recreational purposes (children use them as toys) and 18 considered toxic
Ríos-García et al. [62]	Mazateco	The first ethnomycological study of the Mazatec group is presented, addressing the use, nomenclature, classification, ecology, and gastronomy of 27 species
Ruan-Soto and Ordaz-Velázquez [12]	Maya Lacandon Tsotsil Tseltal Chuj	A bibliographic review of studies in the Maya region revealed 134 edible, 40 medicinal, and 38 toxic mushroom species
Ramírez-Carbajal [19]	Tlahuica	The Tlahuica ethnomycology was described for the first time. The consumption of 160 mushroom species, 79 mushroom names in the indigenous language, and 130 names in Spanish were reported
Servín-Campuzano et al. [20]	Purepecha	Purepechas in the state of Michoacan consume 16 species and have an indigenous name for everyone
Rodríguez-Muñoz et al. [22]	Nahuatl	16 species consumed as food were recorded, and the results indicated that women have the highest knowledge on wild mushrooms
Moreno-Fuentes [24]	Raramuris	Raramuris in the state of Chihuahua use 16 mushroom species as food and 3 as medicine
Haro-Luna et al. [56]	Wixarika Mestizos	37 mushroom species were reported with edible, medicinal, and recreational uses
Cruz-Acevedo [63]	Mazahuas	The use and nomenclature of 78 edible species and 1 toxic species were reported
Mejía-Correa et al. [64]	Totonaca	10 species of cultural importance for food, medicine, and cosmetics purposes were identified
Cipriano-Anastasio et al. [65]	Huasteco	The use of 5 edible mushroom species and 9 names in Spanish were reported
González [66]	Tepehuano	The utilization, nomenclature, classification, ecology, and gastronomy of 14 edible species were explored

The present study documented for the first time the traditional knowledge on wild mushrooms in the Chontal and Chatino groups of Oaxaca, until now it was unknown whether these indigenous groups made use of the fungal resource. Also, the obtained results contributed to increasing the knowledge in the Chinanteco group, which has received little ethnomycological attention. The higher richness of wild edible mushrooms used by Chatinos compared with Chinantecos and Chontales can be explained by the vegetation type they inhabit (transition between vegetation with temperate and tropical affinity), which has been suggested to increase fungal production due to the quantity and quality of resources it provides [31, 67]. However, in different scenarios as the Chinanteco group inhabiting lowlands, the number of mushroom species consumed by people can decrease because tropical environments are likely to provide greater options for natural food resources [68], promoting a varied diet of natural resources consumed by the human groups that inhabit these areas.

Wild mushroom harvesting is an activity in which all members of a family usually participate, as reported in several ethnomycological studies worldwide [69–71], however, there are ethnic groups in Mexico and Africa where men rarely participate in collection [72, 73]. The harvesting of wild mushrooms in the three communities assessed in this study is similar to that reported for the Chinanteco and Lacandon communities in Mexico, and for some rural communities in Cameroon. In these communities, men are responsible for collecting sporomes that grow in places far from the communities, while women and children carry out this activity near the community [60, 74, 75].

Despite the relevance of wild edible mushrooms as non-timber forest products, the various strategies carried out by the Chatino, Chinanteco, and Chontal indigenous groups to conserve areas with high sporome production have been unsuccessful. Several campaigns have been taken to conserve these forests, but the indiscriminate cutting of trees for avocado plantations in the Chontal community and coffee plantations in the Chatino group has accelerated the loss of the original vegetation. Although many ecological studies have shown that agricultural activities and fluctuations in precipitation throughout the rainy season may lead to a critical decline in the availability of wild mushrooms [76–78], the Chinantec people mentioned an increase in the proliferation of edible lignicolous fungi due to agricultural activities where branches and wood debris are left behind.

The names assigned to the mushrooms and the nomenclature used reflect the close relationship between these indigenous groups and the wild mushrooms, demonstrating an ingrained knowledge about fungal resources. The present study showed that, as in all indigenous groups in Mexico, assigning names to wild mushroom species involves understanding their ecological, organoleptic, morphological, and cultural aspects [13, 27, 32, 79]. Contrary to the findings by Berlin [80], who stated that folk classifications lack robust elements to be considered as such, the Chatino and Chontal people assign similar names to taxonomically close species such as *Amanita jacksonii* and *Amanita laurae*, demonstrating their capability to group species into ethnotaxa. This has also been reported in the Yoruba community of Nigeria and various ethnic groups in Mexico. Nevertheless, as observed in certain indigenous groups in Ethiopia, Tanzania, and Mexico, the same species often have distinct names [70, 73].

Wild mushroom classification has been observed to derive from distinctive elements that each indigenous group identifies from a cultural perspective [81]. Nevertheless, *Lactarius volemus* was recorded in the Chatino and Chontal communities and is one of the few species that retains its common name (milk mushroom) in most of the indigenous groups in Oaxaca that have been studied from an ethnomycological perspective, such as Mixtecos, Chinantecos, and Zapotecos [13, 60, 61]. This can be explained by two reasons: (1) the distinctive feature of exuding latex when breaking the gills of the hymenium is a diagnostic element for determining its edibility, and (2) the biocultural richness of the 16 indigenous groups of Oaxaca is the heritage of great cultures that emerged ca. 2900 years ago, such as the Zapotecos and Mixtecos, which facilitated cultural exchange among indigenous groups [82, 83]. This phenomenon converges mainly in the Valles Centrales region of Oaxaca, where species of cultural importance from different regions of the state are represented in several traditional markets [83]. In particular, *Lactarius volemus* has been reported to be sold by people from different communities under the same name in the main markets of Oaxaca [84].

The medicinal use of *Pycnoporus sanguineus* and species from the genus *Psilocybe* has already been documented in previous studies. Mostly for the *Psilocybe* genus, there is a rich tradition of consumption in Mexico, where at least 56 species are known to be used in traditional medicine. In the state of Oaxaca, a total of 31 species have been recorded along with their varieties [30]. The way these mushrooms are used varies within each indigenous group; however, the goal is always to connect people with divine entities and seek guidance regarding different aspects of their lives. The Chinantec people of Santiago Comaltepec and La Esperanza refer to *Psilocybe* species as "dwarf mushrooms" because, after consuming at least four sporomes of *Psilocybe zapotecorum* or *P*. *yungensis* in a healing ceremony, they see a diminutive being who answers questions related to death and illness [60]. Ríos-García et al. [62] reported the use of *P*. *mexicana*, *P*. *caerulescens*, *P*. *cubensis*, and *P*. *yungensis* in nighttime rituals that blend pre-Hispanic and Catholic elements to obtain predictions about future and spiritual healing. The Chatino group reported the use of *P. caerulescens*, *P*. *mexicana*, and *P*. *zapotecorum*, but the method of use is unknown [30, 85].

*Pycnoporus sanguineus* has been reported to be used for the treatment of skin diseases in the Chinanteco community of San Mateo Yetla, Valle Nacional [60], and there are currently several studies on the nutraceutical properties of this species [86]. The medicinal use of wild mushrooms is widespread worldwide [3]. Mexico has an approximate count of 163 medicinal species [87], and the last decade has been of relevance for new records of species used in traditional Mexican medicine [8].

The species that obtained the highest scores for the cultural significance index are *Cantharellus cibarius*, the *Amanita caesarea* complex, *Agaricus pampeanus*, *Ramaria* spp., and *Neolentinus lepideus*. Previous studies conducted in rural communities inhabiting temperate areas of Mexico where the cultural significance index and free-list indices were performed, reported that *Cantharellus cibarius* and the *Amanita caesarea* complex are likely the most culturally significant species in Mexico [13, 53, 88]. However, many indigenous groups have not been studied using this numerical ethnomycological methodology.

Regarding the differential cultural significance of edible wild mushrooms, the Chatino people inhabiting an area with various vegetation types showed that beyond the availability of the fungal resource, taste is the most important parameter in the use of wild mushrooms. This pattern was also observed for the Chinantecos, where the results indicated that the abundance of sporomes does not determine the preference for certain species. However, for the Chontal people who are located in an area with a single vegetation type, abundance and multifunctionality play greater roles in their use of wild mushrooms.

Nevertheless, various studies have found that the preference for certain mushroom species is determined mainly by the amount of biomass they produce [59]. The biomass production in mushrooms together with the ease of recognizing their edibility and multifunctionality, makes them a resource with economic value for several indigenous groups, further enhancing their importance. Under a diverse mosaic of vegetation types, this corresponds to the findings by Ruan-Soto et al. [66], who reported that ecological zones are not the primary element defining the preference for certain wild edible mushroom species. In general, the values obtained for the cultural significance of wild mushrooms consumed in the studied communities exhibited different patterns due to the distinct indigenous groups in which each cultural core perceives and values its natural resources differently [13]. However, despite the three studied indigenous groups being distributed within the state of Oaxaca but with diverse natural landscapes, the existence of regional markets enables broad cultural interchange, reflected in the shared composition of the species they use.

This study was carried out with different age groups to explore generational differences in knowledge acquisition and to recognize how knowledge permeates various scopes and develops in both the worldviews of children and adults in the studied communities. Among children, this knowledge was observed to act as a fundamental guide, transmitted through figures of authority (such as parents and grandparents) and daily practices. Meanwhile, adults not only preserve this knowledge but also incorporate it into their more complex understanding of the environment, as they are mostly in contact with their natural surroundings due to their field activities [89, 90].

The results suggest a loss of traditional knowledge in younger generations caused mainly by the replacement of agricultural and forestry activities for school pursuits and other factors, such as modernization, globalization, and the lack of intergenerational transmission. This loss seems to have affected the preservation of cultural diversity, mostly in the Chatino community where the information obtained and fieldwork activities exposed a lack of interest among young people and children. In the Chontal community, younger generations participate in more forest-related activities, which contributes to their interaction with fungal resources. Similarly, in the Chinanteco group, young people are involved in forest-related activities, albeit to a lesser extent. The generational loss of traditional knowledge has been reported to be strongly related to the decline of mushroom populations worldwide, but also because intoxication caused by the consumption of wild mushrooms makes young people fearful of using this resource [78, 91]. However, as observed in the Chontal and Chinanteco communities, there are ethnic groups where traditional mycological knowledge has persisted in the new generations owing to the stable ethnic identity that comes from strong cultural roots, and the strategies applied to conserve the biocultural heritage [14, 92].

It has been suggested that gender plays a differential role in the acquisition and transmission of mycological knowledge in many cultures [93], corresponding with the findings in the three studied groups where the knowledge of men and women is comparable and both genders are actively involved in the process of harvesting, cooking, and selling mushrooms. This mutual knowledge is likely to come from the strategies used by adults to transmit knowledge to young boys and girls, emphasizing the relevance of becoming involved in fungal resource utilization [14, 69, 94]. In some communities, women play a greater role in the use of fungal resources, as is the case for certain cultural groups in Mexico and Nigeria where the marketing of mushrooms is not a well-remunerated activity, thus, men seek other sources of income [95, 96]. Furthermore, there are ethnic groups where the variation in traditional knowledge between men and women is a result of the division of tasks in the use of this resource, men collect mushrooms and women sell them [9].

Currently, the gap in traditional knowledge among men and women is evolving with gender equality and the promotion of equal opportunities. When the data from the three communities were analyzed together, the obtained results showed that knowledge is being lost among women with higher levels of schooling. This is associated with the fact that more women go to school and leave their communities than men do. Additionally, when women return to their home communities, they engage in small family businesses such as shops, local markets, and food stalls. Men, on the other hand, use to stay in the communities and work in agricultural and forestry activities. However, Berkes et al. [92] and Haro-Luna et al. [14] state that traditional knowledge prevails in societies that make constant use of their natural resources regardless of inhabitants' migration, age, or level of schooling.

In the Chatino group, there was a markedly negative relationship between the level of schooling and the number of mushroom species known by the interviewees, indicating a likely lack of formal education including topics based on their biocultural heritage. Although Mexico's formal education has included several improvements in terms of quality and access to technology, there is still a gap in the null inclusion of traditional knowledge within the educational framework [97], which has generated a deficit in the preservation of cultural richness. This loss of knowledge is also related to the age of the people in the new generations, as little is known in these generations about culturally important mushroom species.

The homogeneous distribution of traditional knowledge in the Chontal and Chinanteco groups regarding the number of known mushroom species concerning the level of schooling, age, and gender is driven by the constant valuation and transmission of traditional knowledge that promotes a scenario of equal opportunities in each of these communities. In addition, they organize workshops and talks that allow interaction between generations, resulting in an exchange of knowledge related not only to wild mushrooms but also to all their biocultural heritage.

Although the Chatino and Chontal groups are geographically closest to each other and shown to share a high richness of mushrooms consumed in their communities, the composition of the species used did not differ conspicuously among the three study groups. Generally, indigenous groups located geographically close to each other share similar wild edible mushroom species, as reported for Mexico and Guatemala, where Tsotsiles, Tzeltales, Mayas, and Lacandones share the use of *Schizophyllum commune*, *Auricularia delicata*, *Cantharellus cibarius*, and *Lactarius indigo* [12]. Additionally, in Central Mexico, Otomies, Nahuatl, Mazahuas, and Purepechas have been reported to consume similar species, such as the *Amanita caesarea* complex, *Laccaria* spp., and *Boletus edulis* [20–22]. This suggests that there is a continuous exchange of traditional knowledge among indigenous groups in Mexico, and the incorporation of new knowledge enriches the culture of ethnic groups.

The mushroom species recorded in the present study have been a staple in the diet of indigenous groups in Mexico for centuries, as they are considered a healthy and essential food for survival during certain seasons of the year [8]. However, it is crucial to raise awareness about cultivable species, such as *Pleurotus djamor* and *Neolentinus lepideus*, which could ensure a year-round availability of nutritious resources and provide an extra economic income in indigenous communities [98], accomplishing the goals outlined in the 2030 Agenda, specifically those related to reducing hunger, poverty, and promoting food security [99].

International and local treaties and laws such as the Convention on Biological Diversity and the General Law of Sustainable Forestry Development, respectively, have emphasized the importance of involving indigenous communities in sustainable forest resource management, and the inclusion of traditional mushroom harvesting practices. [100, 101]. Chontales, Chatinos, and Chinantecos possess valuable knowledge about the mushroom species they use, making it an effective tool for transmitting wisdom regarding the preservation of cultural heritage and the conservation of biodiversity. Therefore, this study's findings can contribute significantly to the formulation of policies that address the utilization of fungal resources in indigenous communities, promoting sustainability, and equity in resource management and conservation.

## Conclusions

Indigenous groups in Mexico are aware that the preservation of traditional knowledge is highly important for continuing to benefit from natural resources. This is evident in the diversity of mushroom species they use, the various practices employed when harvesting, cooking, and consuming the mushrooms, and the extensive information they safeguard regarding this valuable non-timber forest product. The present study enabled the identification of culturally important wild edible mushroom species in three indigenous communities of Oaxaca and showed that factors such as level of schooling and age can be critical for the conservation of traditional knowledge; however, the effects of these factors vary within and across communities. Despite that the results represent a small fraction of the knowledge of these indigenous groups, this study is of great importance because it paves the way for future research on these indigenous groups, which have received limited ethnomycological attention. Conducting studies that encompass a broader range of variables like occupation, migration, and level of expertise in the local language is crucial to gain a comprehensive understanding of the human–mushroom relationship. This approach provides a consensus on how ecological, biological, and cultural factors interrelate in the use and management of culturally significant wild mushrooms. It is essential to promote the documentation and revitalization of these traditions so that future generations can benefit from the wealth of traditional knowledge while embracing modern progress.

### Supplementary Information

**Additional file 1**. Ethnomycological questionnaire. Questionnaire used to obtain information about the biocultural importance of wild mushrooms in the studied indigenous communities.

**Additional file 2**. Macromycete species collected for identification. The file includes species list, voucher numbers, sampling localities, and vegetation types.

**Additional file 3**. Sociocultural information and known species. The data include the age, level of schooling, gender, spoken language (Spanish, indigenous), and the number of known species per interviewee.

## Data Availability

The datasets generated during this study are included in this published article and its supplementary information files.

## Abbreviations

EMCSI: Cultural significance index of wild edible mushrooms
LNI: Local nomenclature index
MI: Mention index
PAI: Perceived abundance index
CFI: Consumption frequency index
MFFI: Multifunctional food index
CPI: Consumption preference index
ERI: Edibility recognition index
EI: Economic index
KTI: Knowledge transmission index
ANOVA: Analysis of variance
ANCOVA: Analysis of covariance
NMDS: Non-multidimensional scaling analysis
HSD: Honestly significant difference
