# Supplementary material for: Variation in traditional knowledge of culturally important macromycete species among three indigenous communities of Oaxaca, Mexico
Source: J Ethnobiol Ethnomed. 2024 Mar 22;20:38. doi: 10.1186/s13002-024-00679-8 (PMC10958891; doi:10.1186/s13002-024-00679-8)

Ethnomycological questionnaire to obtain information about the biocultural importance of wild mushrooms in the studied indigenous groups of Oaxaca, Mexico.

date: ______________

Age:______________________________ gender______________________

Level of schooling: __________________

Speaks Spanish?____________________ Indigenous language: _________________

Occupation: ____

Number of family members:____

1.- Do you know wild mushrooms? Yes ( ) No ( )

2.- What do wild mushrooms mean to you?

3.- Do you know which edible mushrooms grow in the forest?

Name in the indigenous language	Name in Spanish	Meaning and origin of the name

4.- On a scale from 1 to 10, where 1 is the lowest score and 10 is the highest score, how much do you like eating the mushrooms you have mentioned?

1: 2: 3: 4: 5: 6: 7: 8: 9: 10: 11: 12: 13: 14: 15: 16: 17: 18: 19: 20:

5.- How abundant are the mushrooms you have mentioned?"

a) rare b) medium c) common d) very common

1: 2: 3: 4: 5: 6: 7: 8: 9: 10: 11: 12: 13: 14: 15: 16: 17: 18: 19: 20:

6.- How many times do you consume the mentioned mushrooms per season (per year)?

a) Never b) Not every year c) Once a year d) Twice a year e) Three times or more

1: 2: 3: 4: 5: 6: 7: 8: 9: 10: 11: 12: 13: 14: 15: 16: 17: 18: 19: 20:

7.- How do you cook the mushrooms you collect?

Dish name	Mushrooms used

8.- During what months do you harvest edible wild mushrooms?

Name of the mushroom	Harvesting months

9- Where do you harvest wild edible mushrooms?

Name of the mushroom	Growth habit	Vegetation type

10.- How do you identify the mushrooms you consume?"

a) I don't know b) By shape, color, and texture c) By their smell and taste d) By color, shape, taste, and the place where they grow

1: 2: 3: 4: 5: 6: 7: 8: 9: 10: 11: 12: 13: 14: 15: 16: 17: 18: 19: 20:

11.- Do you sell the wild mushrooms that you have harvested?

a) Not for sale b) Occasionally sells them c) Sells them d) Sells them at high prices

1: 2: 3: 4: 5: 6: 7: 8: 9: 10: 11: 12: 13: 14: 15: 16: 17: 18: 19: 20:

12.- In case of selling wild mushrooms, please provide the following information.

Name	Quantity (Kg)	Price ($)	Place where you sell them

13.- In the case of selling mushrooms, does it increase the family income?

14.- What method do you use to collect wild mushrooms?

15.- When you go to collect mushrooms, do you go alone, accompanied by other members of the community, or by your family? How much time do you spend collecting?

16.- Compared to previous years, has mushroom abundance decreased?

17.- Do you know mushrooms with medicinal properties? What is your method of use?

18.- Do you know about other uses given to mushrooms?

19.- Are you aware of any local cases where people have fallen sick due to consuming mushrooms? Do you know which mushroom they ate? When did it happen?

20.- Do you consider of importance the presence of wild mushrooms in a forest? Why?

21.- Are mushrooms still growing in logged areas or areas opened to agriculture? Why?

22.- From whom did you obtain the knowledge you have about wild mushrooms?

a) From an immigrant b) Someone from the same community (not family)

c) Father or mother d) Other member of the family

1: 2: 3: 4: 5: 6: 7: 8: 9: 10: 11: 12: 13: 14: 15: 16: 17: 18: 19: 20:

23.- What is your opinion about your community's current state regarding the loss of traditional knowledge on wild mushrooms?

24.- How do you name in Spanish and indigenous language the parts of a mushroom? (Indicate below).

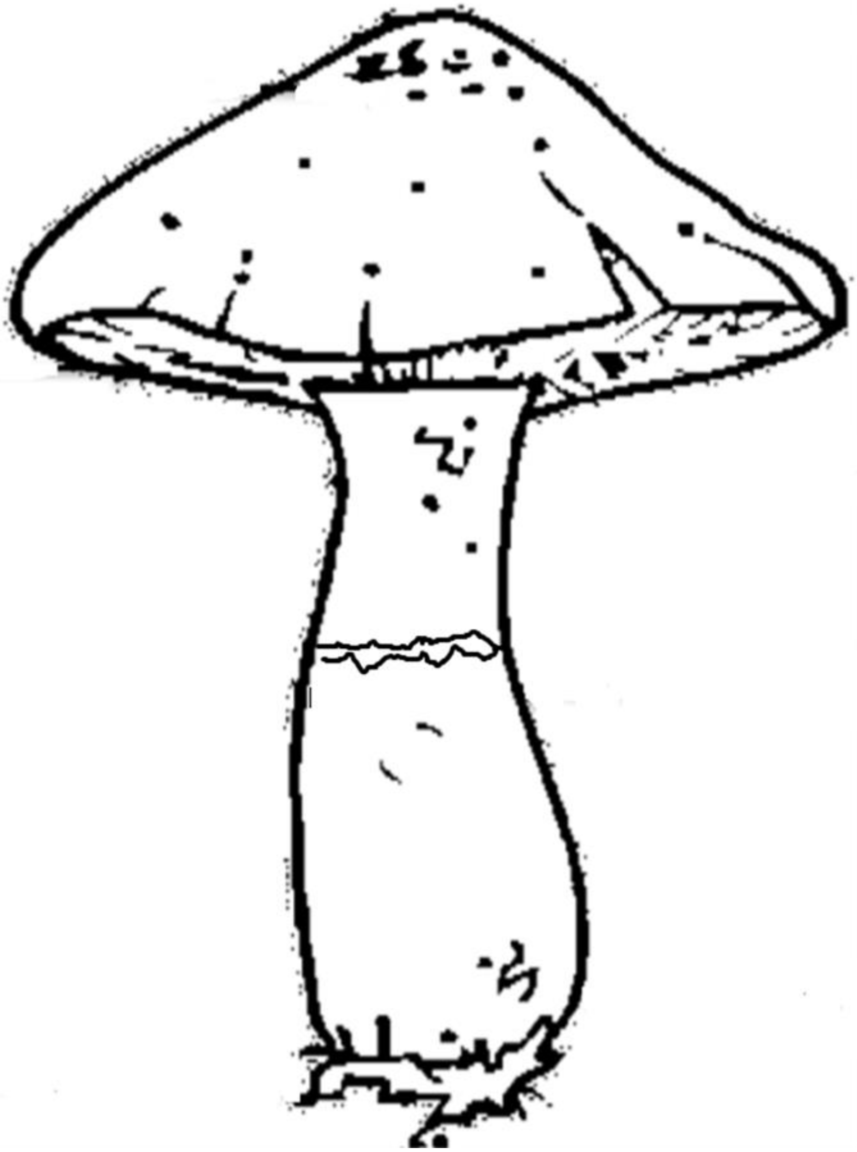

Supplement: Supplementary file 1 — Additional file 1. Ethnomycological questionnaire. Questionnaire used to obtain information about the biocultural importance of wild mushrooms in the studied indigenous communities. [file 13002_2024_679_MOESM1_ESM.pdf]
